# Evolutionary trade-off between heat shock resistance, growth at high temperature, and virulence expression in *Salmonella* Typhimurium

**DOI:** 10.1128/mbio.03105-23

**Published:** 2024-02-13

**Authors:** Daniel Berdejo, Julien Mortier, Alexander Cambré, Malgorzata Sobota, Ronald Van Eyken, Tom Dongmin Kim, Kristof Vanoirbeek, Diego García Gonzalo, Rafael Pagán, Médéric Diard, Abram Aertsen

**Affiliations:** 1 Department of Microbial and Molecular Systems, KU Leuven, Leuven, Belgium; 2 Departamento de Producción Animal y Ciencia de los Alimentos, Facultad de Veterinaria, Instituto Agroalimentario de Aragón-IA2, Universidad de Zaragoza-CITA, Zaragoza, Spain; 3 Biozentrum, University of Basel, Basel, Switzerland; Max Planck Institute for Marine Microbiology, Bremen, Germany

**Keywords:** heat resistance, genetics, evolution, virulence, *Salmonella* Typhimurium

## Abstract

Understanding the evolutionary dynamics of foodborne pathogens throughout our food production chain is of utmost importance. In this study, we reveal that *Salmonella* Typhimurium can readily and reproducibly acquire vastly increased heat shock resistance upon repeated exposure to heat shock. Counterintuitively, this boost in heat shock resistance was invariantly acquired through loss-of-function mutations in the *dnaJ* gene, encoding a heat shock protein that acts as a molecular co-chaperone of DnaK and enables its role in protein folding and disaggregation. As a trade-off, however, the acquisition of heat shock resistance inevitably led to attenuated growth at 37°C and higher temperatures. Interestingly, loss of DnaJ also downregulated the activity of the master virulence regulator HilD, thereby lowering the fraction of virulence-expressing cells within the population and attenuating virulence in mice. By connecting heat shock resistance evolution to attenuation of HilD activity, our results confirm the complex interplay between stress resistance and virulence in *Salmonella* Typhimurium.

**IMPORTANCE:**

Bacterial pathogens such as *Salmonella* Typhimurium are equipped with both stress response and virulence features in order to navigate across a variety of complex inhospitable environments that range from food-processing plants up to the gastrointestinal tract of its animal host. In this context, however, it remains obscure whether and how adaptation to one environment would obstruct fitness in another. In this study, we reveal that severe heat stress counterintuitively, but invariantly, led to the selection of *S.* Typhimurium mutants that are compromised in the activity of the DnaJ heat shock protein. While these mutants obtained massively increased heat resistance, their virulence became greatly attenuated. Our observations, therefore, reveal a delicate balance between optimal tuning of stress response and virulence features in bacterial pathogens.

## INTRODUCTION

*Salmonella enterica* is worldwide among the most notorious zoonotic pathogens, with its *S*. Typhimurium serovar as one of the most commonly reported causes of foodborne illness (1). The virulence of *S*. Typhimurium is mainly directed by the HilD protein, a transcriptional regulator that controls the expression of the *Salmonella* pathogenicity island 1 (SPI-1). SPI-1 encodes a type III secretion system (T3SS) that is required for epithelial invasion and intestinal disease (2). Moreover, next to SPI-1, HilD regulation also impacts (i) the expression of the T3SS-2 encoded on SPI-2, which is essential for *Salmonella* replication and survival within macrophages and the progression of systemic infection (3); (ii) the expression of SPI-4, which encodes a type I secretion system required during the intestinal phase of infection (4, 5); and (iii) motility and chemotaxis (6). Importantly, the HilD regulon is expressed in a bistable fashion (7, 8), leading to a fraction of HilD regulon^OFF^ *S*. Typhimurium cells that remain in a non-invasive state in the gut lumen. There they can profit from inflammation-raised terminal electron acceptors (such as nitrate or tetrathionate) to proliferate via anaerobic respiration and outcompete the endogenous gut microbiome (9, 10). The resulting bloom of *S*. Typhimurium, in turn, supports lateral gene transfer among enterobacteria and the further dissemination to a new host (11). Furthermore, it has been shown that bistable expression of HilD-controlled virulence genes also promotes the formation of an antibiotic-tolerant subpopulation (12).

Next to virulence features required to overtake the host, *S*. Typhimurium also needs to cope with many environmental and manmade stresses. Among these, heat stress is commonly encountered when transiting from the environment to a warm-blooded host or as a result of food preservation measures. In this context, the *S*. Typhimurium heat shock response is well studied and known to be mainly governed by the alternative sigma factor σ^32^ (RpoH) that drives the expression of protective heat shock proteins (HSPs), such as the molecular chaperone systems DnaK/DnaJ/GrpE and GroES/GroEL (13). However, less is known about the evolutionary pathways available for the acquisition of improved heat resistance. One documented evolutionary mechanism entails the acquisition of a genomic island termed the “locus of heat resistance,” which encodes a number of proteins involved in protein quality control (14). In fact, it was previously shown that the notorious heat resistance of *Salmonella* Senftenberg could be attributed to the presence of this island (15, 16). However, a more subtle and unexpected mechanism toward heat resistance acquisition was recently uncovered in *Escherichia coli*, being a close relative of *Salmonella* spp. Here, it was shown that mutations in the *E. coli tnaA* gene (encoding the tryptophanase enzyme) could lead to the production of folding-compromised TnaA variants that, in turn, pre-emptively boost the expression of HSPs to higher basal levels (17).

In this study, we reveal that *S*. Typhimurium could readily and reproducibly acquire increased heat resistance when it is exposed to recurrent lethal heat stress. Surprisingly, however, this heat resistance was invariably caused by loss-of-function mutations in the *dnaJ* gene that encodes the molecular chaperone DnaJ. As a result, the acquisition of heat shock resistance counterintuitively coincided with lower maximal growth temperatures. Moreover, the loss of DnaJ also compromised the activity of HilD, which reduced the virulence of *S*. Typhimurium in mice.

## RESULTS

### *S*. Typhimurium can readily acquire strongly improved heat shock resistance

**Fig 1:**
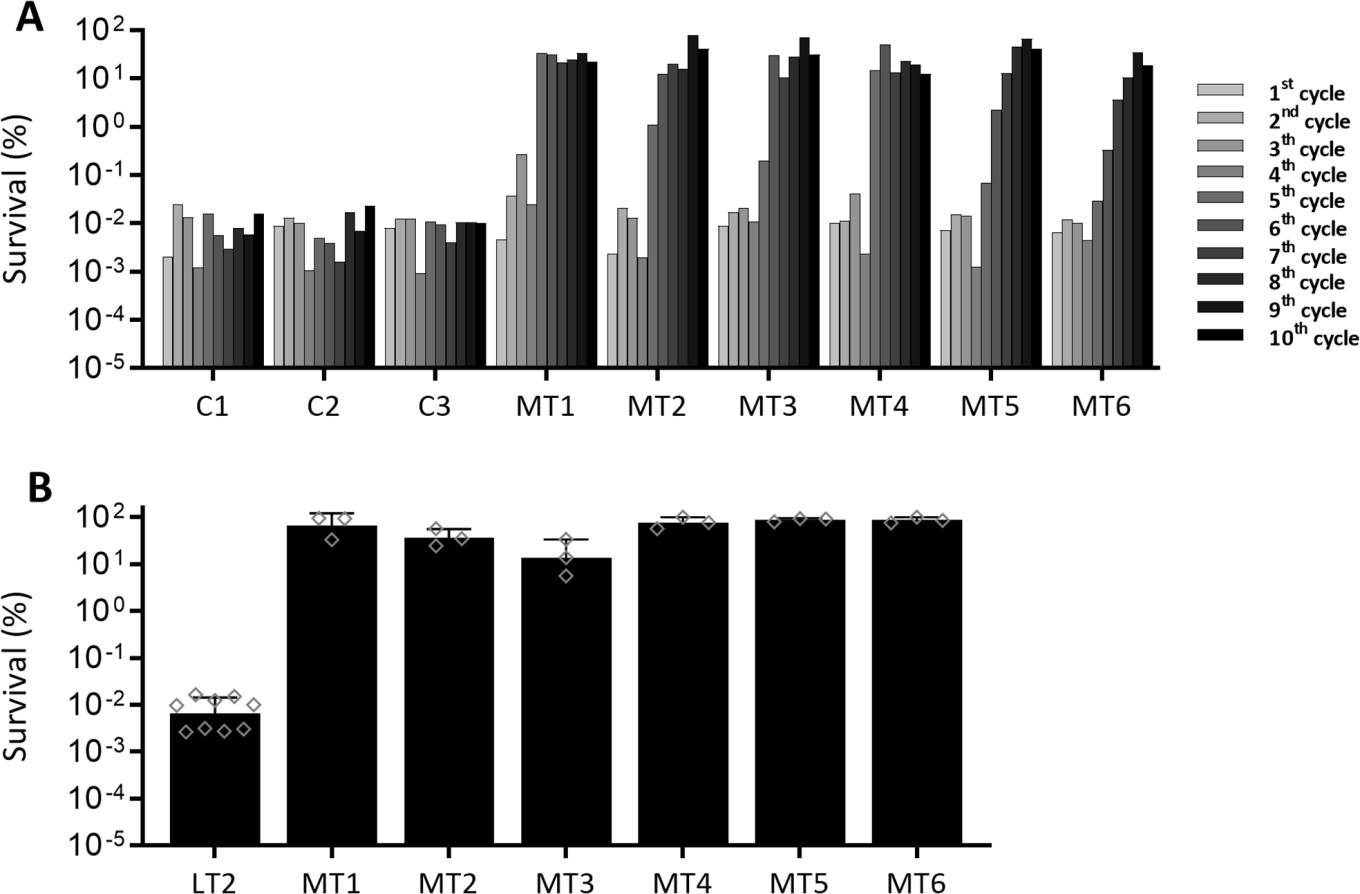
Directed evolution of *S. enterica* Typhimurium LT2 toward heat resistance. Six independent axenic cultures of *S. enterica* Typhimurium LT2 (MT1, MT2, MT3, MT4, MT5, and MT6) were iteratively exposed to the same heat treatment (55°C for 20 min), with a resuscitation and growth step (ca. 24 h in total) in TSB between consecutive treatments, until 10 cycles were completed (**A**). The same protocol was applied for three independent axenic cultures of *S. enterica* Typhimurium LT2 without heat stress during the cycles as experimental controls (**C1, C2, and C3**). At the end of the evolution assays, heat resistance (**B**) was evaluated under the same treatment conditions (55°C for 20 min) for the parental LT2 stain and a representative clone isolated from each of the six independently evolved lineages (i.e., clone MT1-6) grown to stationary phase for ca. 24 h in TSB. Survival was expressed as percentage (%) of the viable cells after the treatment with respect to the initial population (set at 100%), as determined by plate counts. For panel A, a single replicate was performed for each cycle for all the lineages. For panel B, individual data points are plotted as diamonds and bars display the means with the standard deviations over at least three independent experiments.

In order to probe the potential for heat shock resistance development in *S*. Typhimurium strain LT2 (further referred to as LT2), a directed evolution approach was followed. When iteratively exposing six independent lineages of LT2 in stationary phase to a heat shock (55°C for 20 min) with intermittent resuscitation and outgrowth of survivors, we were surprised to note that each of these lineages rapidly acquired a massive >1,000-fold resistance compared to the three control-cycled lineages (i.e., serially passaged in the absence of intermittent heat stress) (Fig. 1A). Upon purification, randomly isolated clones of each heat-exposed lineage (designated MT1-MT6), indeed, confirmed their ca. 1,000-fold heat shock resistance compared to the parental LT2 strain or clones isolated from the control-cycled lineages (Fig. 1B).

### Loss of DnaJ functionality underlies rapidly acquired heat shock resistance in *S*. Typhimurium

**Fig 2:**
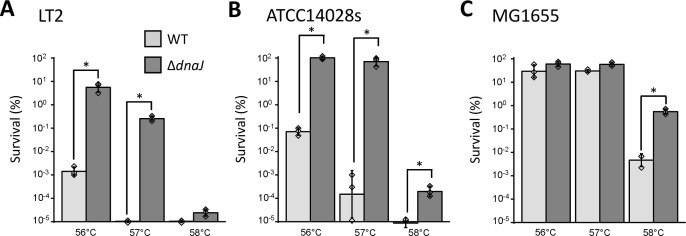
Survival after heat exposure (56°C, 57°C, or 58°C for 15 min) of (**A**) *S*. Typhimurium LT2 wild type (light gray) and its Δ*dnaJ* derivative (dark gray), (**B**) *S*. Typhimurium ATCC14028s wild type (light gray) and its Δ*dnaJ* derivative (dark gray), and (**C**) *E. coli* MG1655 Δ*lacY* wild type (light gray) and its Δ*dnaJ* derivative (dark gray) grown to stationary phase for ca. 18 h in TSB. Survival was expressed as percentage (%) of the viable cells after the treatment with respect to the initial population (set at 100%), as determined by plate counts (detection limit 200 CFU/mL). The displayed means were determined over three independent experiments and the error bars indicate the standard deviation over these experiments. The individual data points are plotted as diamonds. Asterisks indicate statistically significant differences (ANOVA followed by Tukey HSD *post-hoc* test, *P*-value ≤ 0.05).

Surprisingly, whole-genome sequencing of these six independently evolved heat shock-resistant LT2 clones (i.e., MT1–6) revealed that all of them incurred mutations in the *dnaJ* gene, while four of them also incurred mutations in either the *flhC* or *fliF* flagellar genes (Table 1). In contrast, the *dnaJ*, *flhC,* and *fliF* genes remained unaltered in the control-cycled clones (as confirmed with Sanger sequencing). Since most of the *dnaJ* mutations appeared to be frame-shifting loss-of-function mutations (Fig. S1), a *de novo* deletion of *dnaJ* was synthetically reconstructed in the LT2 parent and subsequently heat challenged. This confirmed that loss of DnaJ functionality was, indeed, causative and on itself sufficient for the increased heat shock resistance, conferring a ca. 10,000-fold increased survival compared to the wild-type (WT) LT2 strain when exposed to 56°C or 57°C for 15 min (Fig. 2A).

**TABLE 1:** Mutations of indicated evolved heat shock-resistant LT2 clones in comparison with LT2, as verified by Sanger sequencing^*c*^

Resistant LT2 clones	Genome position^*a*^	Gene	Locus tag	Mutation^*b*^	Change	Information
MT1	14,209	*dnaJ*	STM0013	Dup: 616_623 CATGGGCG	Frameshift variant (Val209)	Chaperone protein DnaJ
	1,335,493	*–*	STM1250-STM1251	SNV: C5A	Intergenic region	In between STM1250 and STM1251
	3,583,355	*rpoA*	STM3415	SNV: G943A	Gly315Ser	DNA-directed RNA polymerase subunit alpha
MT2	14,250	*dnaJ*	STM0013	Del: 657_670 CCCGGCGGGCGTGG	Frameshift variant (Pro220)	Chaperone protein DnaJ
	2,021,338	*flhC*	STM1924	SNV: G380A	Arg127His	Regulator of flagelar biosynthesis
MT3	14,689	*dnaJ*	STM0013	Dup: 1101_1119 TGACGGCGTGAAAAAATTC	Frameshift variant (Phe367)	Chaperone protein DnaJ
	2,057,925	*fliF*	STM1969	Del: 1390_1396 CGCTGGT	Frameshift variant Arg464	Flagellar M-ring protein FliF
	3,376,290	*rpoD*	STM3211	SNV: G18T	Silent mutation (Arg6)	RNA polimerase sigma factor rpoD
MT4	13,776	*dnaJ*	STM0013	Del: A186	Frameshift variant (Lys62)	Chaperone protein DnaJ
	2,021,276	*flhC*	STM1924	SNV: A442C	Thr148Pro	Regulator of flagelar biosynthesis
MT5	14,251	*dnaJ*	STM0013	Del: 664_679 GGCGTGGATACCGGCG	Frameshift variant (Gly222)	Chaperone protein DnaJ
	2,021,224	*flhC*	STM1924	SNV: C494T	Pro165Leu	Regulator of flagelar biosynthesis
MT6	14,409	*dnaJ*	STM0013	SNV: T815G	Ile272Ser	Chaperone protein DnaJ
	2,372,274	*rcsC*	STM2271	SNV: T1319C	Ile440Thr	Two-component system histidinekinase/response regulator

^*a*^ NCBI accession number: NC_003197.2.

^*b*^ Position with respect to the start of the coding region.

^*c*^ Single-nucleotide variation (SNV), duplication (Dup), deletion (Del), and insertion (Ins).

Since the LT2 strain has been documented to harbor an attenuated *rpoS* allele (18) that might make it hypersensitive to inactivation by heat shock, we also synthetically deleted *dnaJ* in the *S*. Typhimurium ATCC14028s strain that harbors an intact *rpoS* allele. In this background, loss of DnaJ even imposed 1,000- to 1,000,000-fold heat shock resistance when exposed to 56°C or 57°C for 15 min (Fig. 2B).

To further investigate whether this DnaJ-mediated phenomenon was specific for *S*. Typhimurium, *dnaJ* was synthetically deleted in an *E. coli* K12 MG1655 background [i.e., MG1655 Δ*lacY*; constructed in reference (19)]. Although this background displayed higher resistance to heat shock compared to *S*. Typhimurium, loss of DnaJ also caused ca. 100-fold increased resistance when exposed to 58°C for 15 min (Fig. 2C).

**Fig 3:**
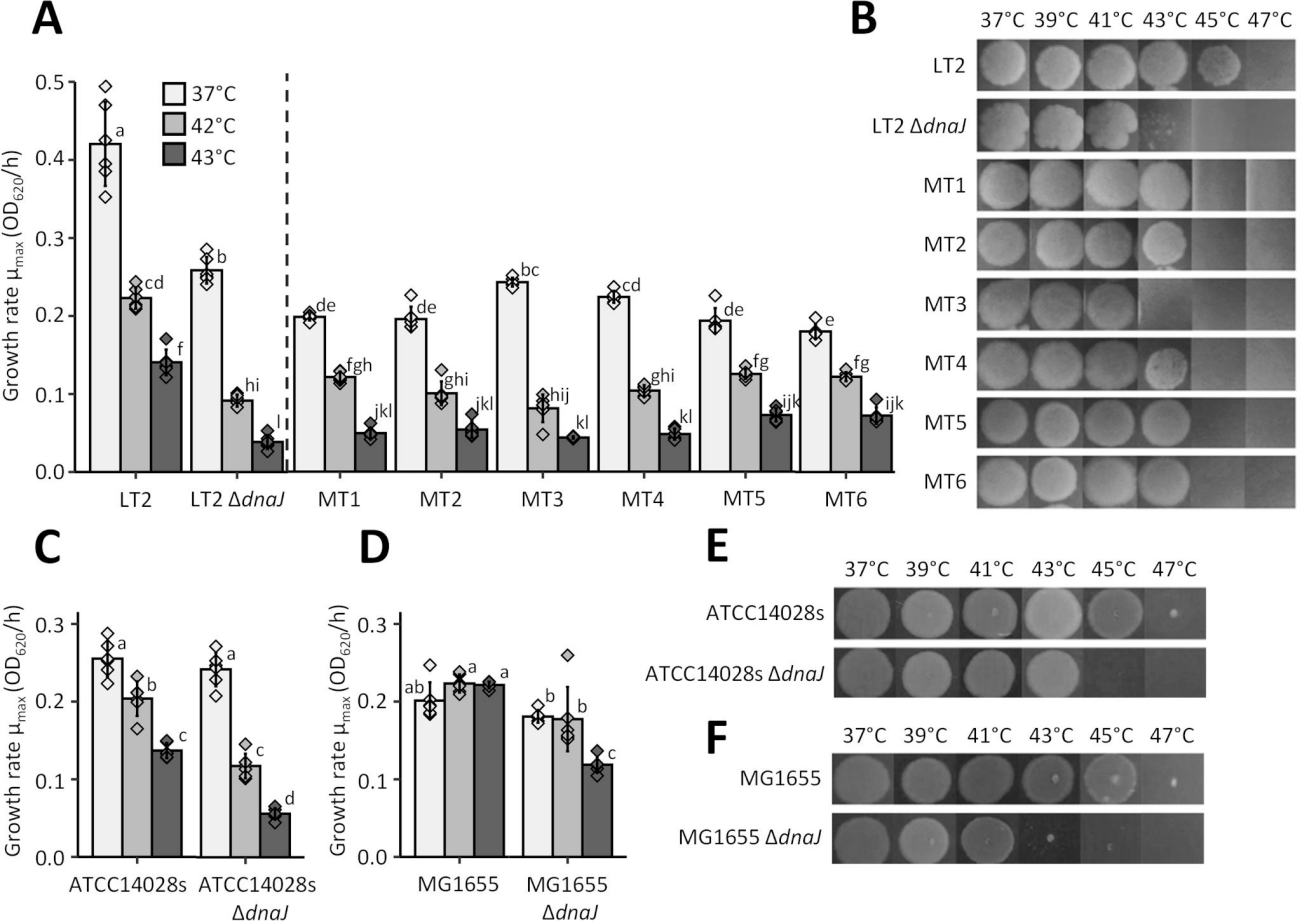
(**A**) Estimated maximum growth rates (*µ*_max_) based on OD_620_ measurements of wild-type LT2, its Δ*dnaJ* derivative, and the heat-selected mutants (MT1–6) grown for 12 h in TSB microtiter plates at 37°C (light gray), 42°C (middle gray), or 43°C (dark gray). (**B**) Growth assay of wild-type LT2, its Δ*dnaJ* derivative, and the heat-selected mutants (MT1–6) grown at the indicated temperatures for 24 h after spotting 5 µL of a 1/1,000 diluted overnight TSB culture on TSA plates. (**C**) Estimated maximum growth rates (*µ*_max_) based on OD_620_ measurements of ATCC14028s and its Δ*dnaJ* derivative grown for 12 h in TSB microtiter plates at 37°C (light gray), 42°C (middle gray), or 43°C (dark gray). (**D**) Estimated maximum growth rates (*µ*_max_) based on OD_620_ measurements of *E. coli* MG1655 Δ*lacY* and its Δ*dnaJ* derivative grown for 12 h in TSB microtiter plates at 37°C (light gray), 42°C (middle gray), or 43°C (dark gray). (**E**) Growth assay of ATCC14028s and its Δ*dnaJ* derivative grown at the indicated temperatures for 24 h after spotting 5 µL of a 1/1,000 diluted overnight TSB cultures on TSA plates. (**F**) Growth assay of *E. coli* MG1655 Δ*lacY* and its Δ*dnaJ* derivative grown at the indicated temperatures for 24 h after spotting 5 µL of a 1/1,000 diluted overnight TSB cultures on TSA plates. For panels A, C, and D, the individual data points are plotted as diamonds, and within a panel, different letters indicate statistically significant differences among different strains and growth temperatures (ANOVA followed by Tukey HSD *post-hoc* test, *P*-value ≤ 0.05), and error bars indicate the standard deviation over six separately grown cultures. For panels B, E, and F, a representative picture of three independent experiments is shown.

As an important trade-off, and in contrast to the heat shock resistance, loss of DnaJ severely attenuated growth at higher temperatures (Fig. 3; Fig. S2). In fact, LT2 Δ*dnaJ* displayed highly compromised growth kinetics (i.e., a ca. fourfold reduction in maximum growth rate) compared to the LT2 wild type at 43°C and even already showed a ca. twofold reduction in maximum growth rate at 37°C (Fig. 3A; Fig. S2). When testing growth over a wider range of temperatures, we found that all the tested Δ*dnaJ* strains lost the ability to form colonies (after a 24-h incubation period) around 45°C (Fig. 3B, E and F). When looking at the heat-selected LT2 mutants with spontaneous *dnaJ* mutations (i.e., MT1-6), all of them were likewise compromised in growth at 37°C and higher temperatures compared to the parental strain (Fig. 3A and B; Fig. S2).

### Loss of DnaJ functionality attenuates virulence of *S*. Typhimurium

**Fig 4:**
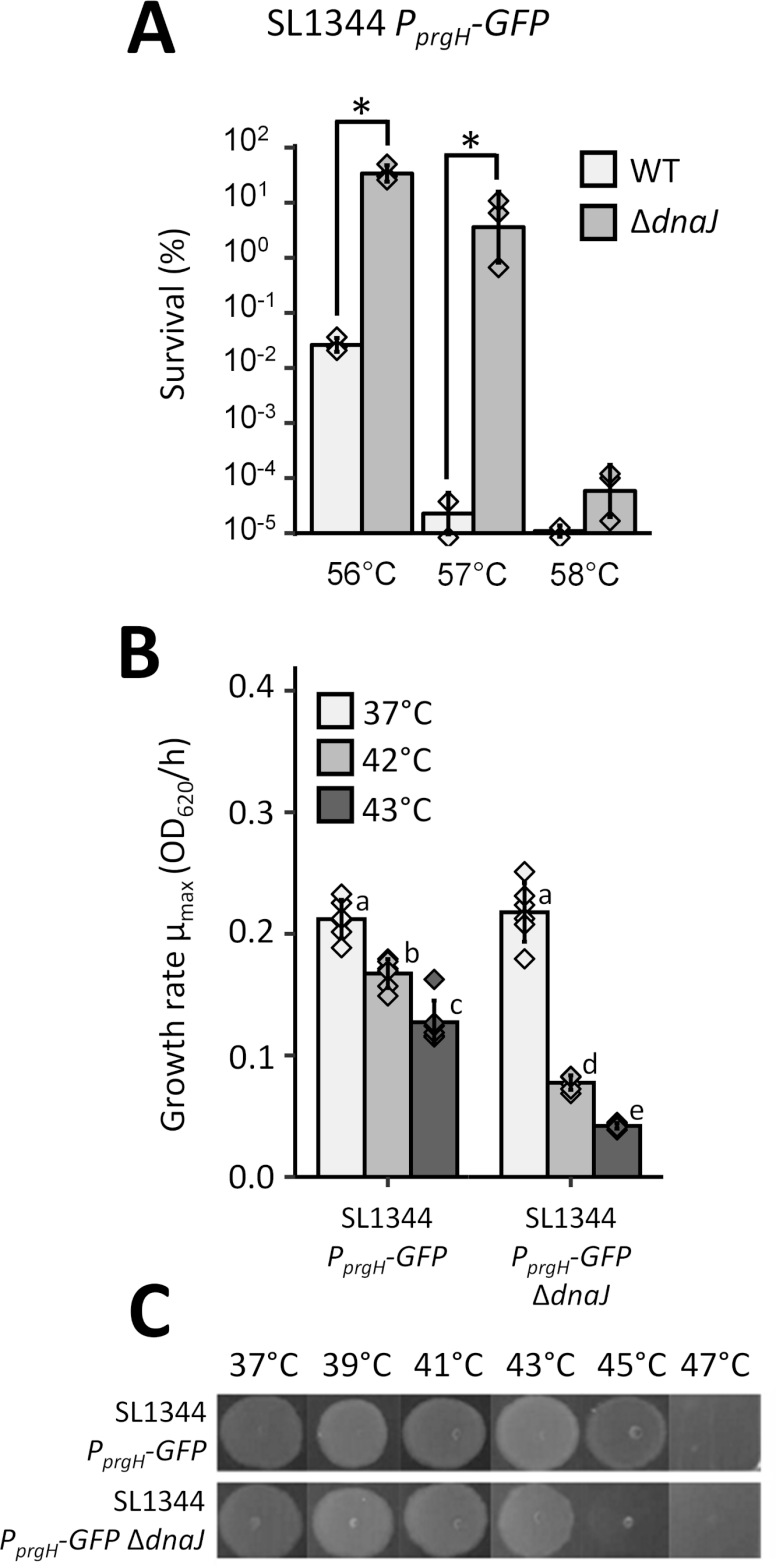
(**A**) Survival after heat exposure (56°C, 57°C, or 58°C for 15 min) of *S*. Typhimurium SL1344 *P_prgH_-GFP* wild type (WT; light gray) and its Δ*dnaJ* derivative (dark gray) grown to stationary phase for ca. 18 h in TSB. Survival was expressed as percentage (%) of the viable cells after the treatment with respect to the initial population (set at 100%), as determined by plate counts (detection limit 200 CFU/mL). The displayed means were determined over three independent experiments, and the error bars indicate the standard deviation over these experiments. The individual data points are plotted as diamonds. Asterisks indicate statistically significant differences (ANOVA followed by Tukey HSD *post-hoc* test, *P*-value ≤ 0.05). (**B**) Estimated maximum growth rates (*µ*_max_) based on OD_620_ measurements of *S*. Typhimurium SL1344 *P_prgH_-GFP* and its Δ*dnaJ* derivative grown for 12 h in TSB microtiter plates at 37°C (light gray), 42°C (middle gray), or 43°C (dark gray). The individual data points are plotted as diamonds, different letters indicate statistically significant differences among different strains and growth temperatures (ANOVA followed by Tukey HSD *post-hoc* test, *P*-value ≤ 0.05), and error bars indicate the standard deviation over six separately grown cultures. (**C**) Growth assay of *S*. Typhimurium SL1344 *P_prgH_-GFP* and its Δ*dnaJ* derivative grown at the indicated temperatures for 24 h after spotting 5 µL of a 1/1,000 diluted overnight TSB cultures on TSA plates. A representative picture of three independent experiments is shown.

Since the interplay between stress resistance and virulence is often neglected but highly relevant in foodborne pathogens, we examined the impact of DnaJ deprivation on the HilD regulon. More specifically, *dnaJ* was synthetically deleted in the well-characterized *S*. Typhimurium SL1344 *P_prgH_-GFP* reporter strain in which GFP fluorescence is directly related to HilD activity via the SPI-1-borne *prgH* promoter (*P_prgH_*) (20). Similar to the LT2 and ATCC14028s backgrounds described above, loss of DnaJ caused SL1344 *P_prgH_-GFP* to become 1.000- to 100.000-fold resistant to a 56°C or 57°C heat shock (Fig. 4A) but sensitive to growth at elevated temperatures (Fig. 4B and C; Fig. S3).

**Fig 5:**
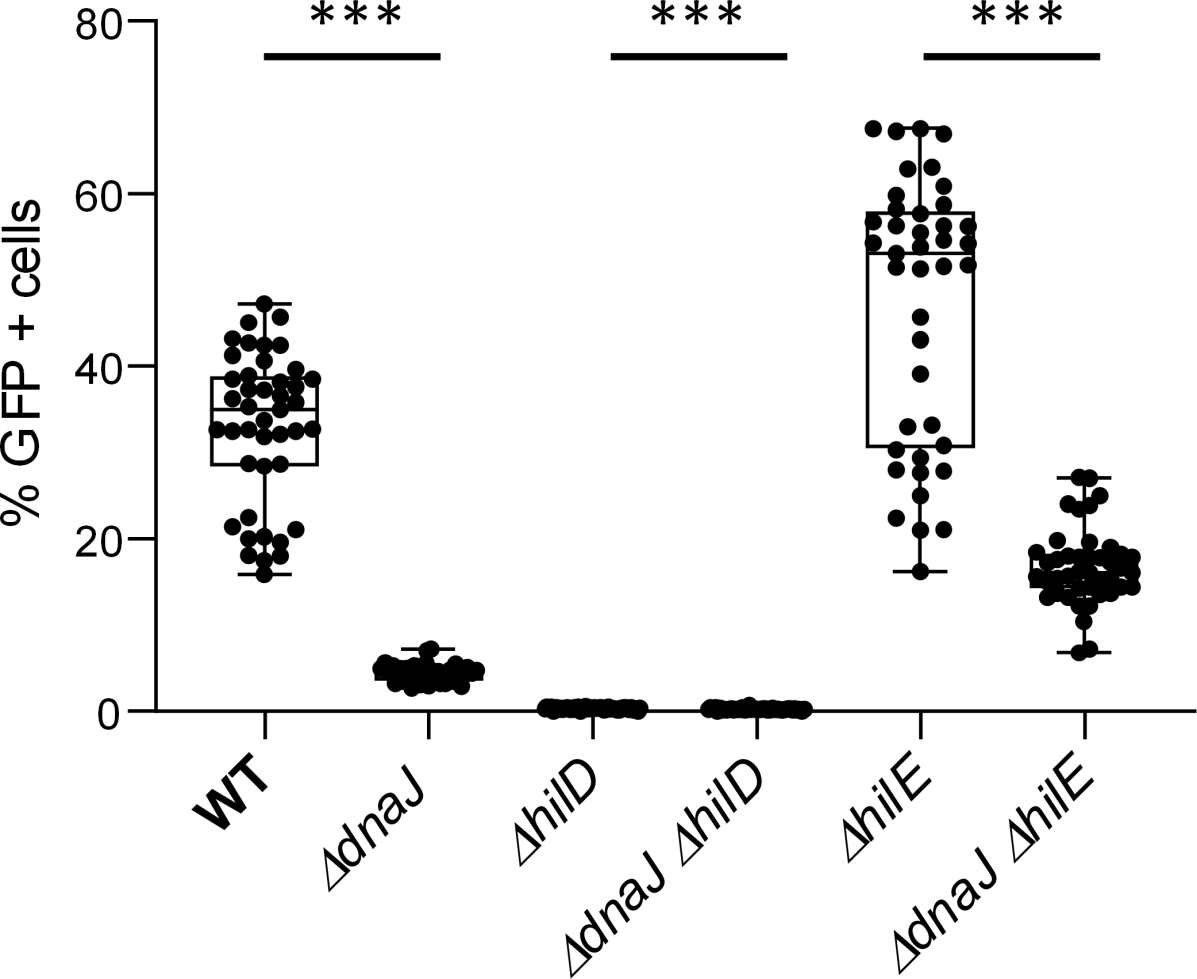
Expression of virulence at the single-cell level of *S*. Typhimurium SL1344 P*_prgH_-GFP* WT and its indicated deletion mutants derivative strains including *ΔdnaJ* mutants. Within a boxplot, each dot represents an independent population of which the proportion of P*_prgH_-GFP* expressing (i.e., GFP^+^) cells was determined with flow cytometry. Wilcoxon matched-pairs signed rank test *P* < 0.001 (***). Outliers were identified using the ROUT method (21) with *Q* = 1%. Statistical significance was assessed using the data devoid of the outliers via Wilcoxon matched-pairs signed rank test.

Subsequently, since HilD activity is intrinsically bimodally distributed throughout a clonal population (7, 8), the relative proportion of GFP expressing (i.e., HilD regulon^ON^) cells was compared between the Δ*dnaJ* and parental reporter strain. This surprisingly revealed that loss of DnaJ severely reduced the proportion of HilD regulon^ON^ cells (Fig. 5). In fact, while the parental SL1344 *P_prgH_-GFP* population typically contains ca. 33% of HilD regulon^ON^ cells, this became ca. 4.5% in the Δ*dnaJ* mutant. Moreover, even in a Δ*hilE* background (in which the fraction of HilD regulon^ON^ cells is genetically upregulated), the loss of DnaJ reduced the fraction of HilD regulon^ON^ cells. Please note that a Δ*hilD* mutant abolishes *P_prgH_* expression (resulting in almost no HilD regulon^ON^ cells) and was, therefore, included as a control of the reporter.

**Fig 6:**
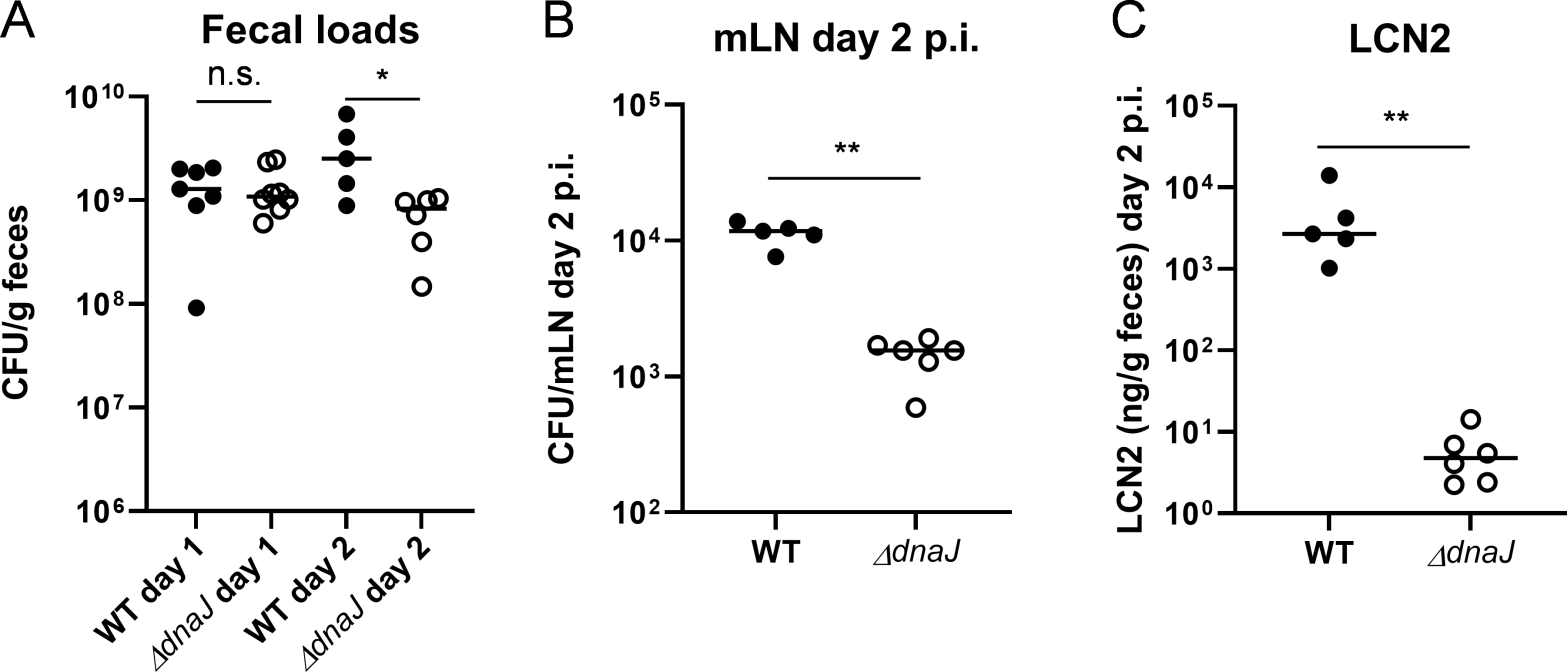
Comparison of virulence in mice between *S*. Typhimurium SL1344 P*_prgH_-GFP* WT and its *ΔdnaJ* derivative. Streptomycin-pretreated mice were infected orally by *S*. Typhimurium SL1344 P*_prgH_-GFP* WT (*n* = 5) or its *ΔdnaJ* derivative (*n* = 6) and sacrificed at day 2 post-infection (p.i.). (**A**) Fecal *S*. Typhimurium loads at days 1 and 2 p.i., (**B**) *S*. Typhimurium loads in mesenteric lymph nodes (mLN) at day 2 p.i., and (**C**) intestinal inflammation at day 2 p.i. revealed by lipocalin-2 (LCN2) dosage in feces samples. Unpaired Mann-whitney test *P* < 0.05 (*); *P* < 0.01 (**); n.s., non-significant.

Subsequent infections in mice accordingly revealed that loss of DnaJ severely attenuated the virulence of SL1344. More specifically, while the *dnaJ* mutant was able to colonize the large intestine of antibiotic pretreated mice (Fig. 6A), it was unable to infect the mesenteric lymph nodes (Fig. 6B) and did not trigger acute inflammation (Fig. 6C). This is in line with a previous observation that *dnaK* deletion (encoding a co-chaperone for DnaJ) causes reduced virulence of *S.* Typhimurium in mice (22)

## DISCUSSION

Our data indicate that a readily acquired loss-of-function mutation in *dnaJ* can dramatically improve *S*. Typhimurium resistance to heat shock (i.e., 1,000- to 100,000-fold, depending on the strain and the temperature). Furthermore, a similar effect occurs in *E. coli* as well. These are counterintuitive findings revealing that loss of a HSP can actually be an evolutionary path to increase heat resistance. However, in all backgrounds tested, the increased ability to survive and recover from a heat shock (i.e., short exposure to lethal temperatures) coincided with attenuated growth at 37°C and an inability to sustain growth at temperatures above 43°C (for *S*. Typhimurium) or even above 41°C (for *E. coli*). This unexpected discrepancy seems to hint at a previously unrecognized evolutionary decoupling between the requirements for heat shock resistance and those for growth at higher temperature.

DnaJ acts as a co-chaperone of DnaK and is involved in the folding of nascent polypeptides, refolding of misfolded proteins, and disaggregation of protein aggregates (23–25). Importantly, previous studies have shown that DnaJ also plays a central role in stimulating DnaK to bind the sigma-factor σ^32^ (RpoH) and allow downregulation of the heat shock response by the inactivation and destabilization of σ^32^ and its degradation by the FtsH protease (26–30). It is likely that either increased protein misfolding due to loss of DnaJ or alleviation of the negative control by DnaKJ on σ^32^ leads to increased basal levels of HSPs and a subsequent increase in heat shock resistance in *S*. Typhimurium. In fact, it has previously been demonstrated in *E. coli* that lack of DnaJ results in higher basal levels of HSPs and a delayed shut-off of the heat shock response due to compromised inhibition of σ^32^ (28). Counterintuitively, this mechanism also suggests that—unlike heat shock resistance—growth at higher temperatures is severely compromised by higher basal levels of HSPs and/or by the lack of DnaJ functionality.

Although our results indicate that loss of DnaJ also provides considerable heat resistance in *E. coli*, *dnaJ* mutations were so far not detected in similar directed evolution approaches toward heat resistance in *E. coli* (17). In contrast, *E. coli dnaJ* mutants can be selected after exposure to phage Lambda since the phage requires DnaJ for the destabilization of the Lambda phage P-DnaB bound to the *ori* site to allow unwinding and replication of DNA (31–33). In turn, this implies that selection pressures imposed by phage predation could simultaneously select for unrelated features such as increased heat resistance. With the rise of phage therapy to control bacterial pathogens in agriculture, food production, and medicine, such confounding dynamics might need closer attention. Rather than *dnaJ* mutations, recent research in *E. coli* has shown that evolution toward heat resistance could select for subtle gain-of-function mutations in the gene-encoding tryptophanase (*tnaA*) (17). More specifically, these mutations lead to folding-compromised TnaA variants that are able to pre-emptively boost the expression of HSPs to higher basal levels (17), thereby causing improved heat resistance.

Regardless of the fact that this particular TnaA-dependent evolutionary path is blocked in *S*. Typhimurium (since *Salmonella* spp. naturally lack the *tna* operon), the DnaJ-dependent route might be selected because of an additional benefit to the heat resistance of *S*. Typhimurium. In fact, we have recently shown that the production of virulence factors in *S*. Typhimurium leads to increased membrane instability and heat shock sensitivity (20). As such, next to HSP upregulation, the specific decrease in HilD-mediated virulence expression we observed in the Δ*dnaJ* mutant might contribute to heat shock survival as well. It was previously observed in *S*. Typhimurium that loss of DnaK or genetic upregulation of σ^32^ activity leads to accumulation of the Lon protease that, in turn, progressively degrades the HilD regulon (34), and loss of DnaJ most likely acts along the same line. In the same vein, Table 1 also reveals that heat selected *S*. Typhimurium mutants regularly incur mutations in flagellar regulation or biosynthesis (i.e., mutations in *flhC* or *fliF*), which is in line with flagella being identified as the virulence factor with the highest cost on stress resistance (20).

Our results indicate that acquisition of heat resistance can readily and reproducibly lead *S*. Typhimurium to lose its DnaJ functionality, which, in turn, has immediate consequences on the maximal growth temperature, and virulence of this pathogen. This underscores that DnaJ is at the intersection of these important features and that evolutionary modulation of its functionality can navigate the trade-offs between growth and resistance and resistance and virulence.

## MATERIALS AND METHODS

### Strains and growth conditions

The bacterial strains and plasmids used in this study are listed in Table S1, and primers are listed in Table S2. For liquid culturing of bacteria, Tryptone Soy Broth (TSB) was used at 37°C and incubated aerobically with shaking (250 rpm) in tubes containing 4 mL of medium. Stationary phase cultures were obtained by incubation for ca. 18 or 24 h (as indicated). For culturing on solid medium, Tryptone Soy Agar (TSA) was used.

### Heat shock treatment of liquid cultures

For heat treatment, stationary phase cultures were harvested by centrifugation (6,000 × *g*, 5 min) and resuspended in an identical volume of 0.85% KCl. Subsequently, a PCR tube containing 50 µL of the resuspended culture was heat treated at the indicated temperature and time in a Biometra T3000 Thermocycler (Biometra, Göttingen, Germany). Unstressed control cultures were simultaneously kept at room temperature for the duration of the treatment. Survival was determined by aseptically retrieving the treated cultures from the PCR tubes, serially diluting heat stressed and unstressed cultures in 0.85% KCl and spotting 5 µL drops onto Tryptone Soy Agar (TSA; Oxoid) as previously described (35). After 24 h of incubation at 37°C, the CFU/mL was determined by counting colonies in spots containing between 5 and 50 colonies. The limit of quantification corresponds to 200 CFU/mL. Finally, survival was expressed as percentage (%) of the viable cells after the treatment with respect to the initial population (set at 100%).

### Selection for heat shock resistance

Heat-resistant mutants were obtained by repeatedly subjecting six independent axenic cultures of LT2 to heat shocks of 55°C for 20 min, with intermittent resuscitation and outgrowth of the survivors. After each heat shock, the heat-treated samples were diluted 1/100 in fresh TSB and subsequently grown for ca. 24 h at 37°C in order for the survivors to resuscitate and grow to stationary phase before the next round of heat treatment. Additionally, a number of independent control-lineages were subjected to the same regime in the absence of heat stress in order to determine the potential selective effect of serially passing through TSB. Survival was determined after each round, and after nine rounds of selection, a single colony from each of the six heat-selected lineages was purified on TSA to obtain a representative clone for whole-genome sequencing (see below). Each of the obtained clones and the parental LT2 strain were grown for ca. 24 h in TSB and subjected to a heat shock of 55°C for 20 min, after which survival was determined and compared to confirm the heat resistance of the isolated clones (i.e., MT1-6).

### Whole-genome sequencing

Genomic DNA was isolated from overnight LB cultures of LT2 and MTs strains using the GeneJET genomic DNA purification kit (Thermo Fisher Scientific, Waltham, MA, USA), after which 150 bp paired-end libraries were prepared using the Nextera DNA Flex library prep kit (Illumina, San Diego, CA, USA) and Nextera DNA CD indexes (set of 24 indexes) (Illumina). Sequencing was carried out with an Illumina MiniSeq sequencer using a MiniSeq Mid Output Kit (300-cycles) (Illumina, San Diego, CA, USA) and analyzed with QIAGEN CLC Genomics Workbench 12.0.3 (Qiagen, Aarhus, Denmark, https://digitalinsights.qiagen.com/). The sequencing reads of our LT2 wild-type strain were trimmed and mapped to the NCBI reference genome (LT2, NCBI accession number: NC_003197.2) to generate a reference consensus sequence of our own wild-type LT2 strain. The sequencing reads of the mutant strains were then trimmed and mapped to this newly made reference LT2 genome and analyzed for single-nucleotide polymorphisms (SNPs, via basic variant detection command), indels (InDels, via InDels, and structural variants command), and structural variants (SVs, via InDels, and structural variants command). All mutations detected by WGS were verified by Sanger sequencing.

### Mutant construction

The deletion of *dnaJ* was performed according to the method of Datsenko and Wanner (36) by creating an amplicon containing a kanamycin resistance cassette on pKD13 with primer pairs P1/P2 (LT2), P5/P6 (ATCC14028s), P9/P10 (MG1655), and P13/P14 (SL1344) (Table S2). This amplicon was recombineered after the start codon of *dnaJ* in a pKD46-equipped strain. In the *E. coli* MG1655 background, the *frt*-flanked kanamycin resistance cassette was excised by transiently equipping the strain with the plasmid pCP20 [expressing the Flp recombinase; (37)]. Despite multiple attempts of excising the kanamycin resistance gene in the *S*. Typhimurium LT2, ATCC14028s, and SL1344 backgrounds, these attempts remained unsuccessful. All constructed mutants were verified by sequencing (Macrogen, Amsterdam, the Netherlands) using primers that anneal upstream and downstream of the *dnaJ* locus (Table S2).

### Growth assays

Cells from a 18 h-grown stationary phase TSB culture were diluted 1/100 in 200 µL of fresh TSB medium in a 96-well microplate (Greiner Bio-One) and subsequently grown aerobically in a Multiskan FC (Thermo Fisher Scientific, Waltham, MA, USA) for 12 h at 37°C, 42°C, or 43°C. The optical density (OD) was automatically measured at 620 nm every 15 min. Growth curves were analyzed by fitting the growth model described by Baranyi and Roberts (38) using the DMFit 3.5 software (Institute of Food Research, Norwich Research Park, Norwich, United Kingdom) to obtain the maximal growth rate *µ*_max_.

Alternatively, growth was assessed by diluting 18 h-grown stationary phase TSB cultures 1/1,000 in fresh TSB and spotting 5 µL drops of this suspension on TSA plates followed by 24 h of incubation of 37°C, 39°C, 41°C, 43°C, 45°C or 47°C.

### Expression of virulence measured at single cell level by flow cytometry

Late exponential phase cultures (OD = 0.8) in LB harboring the P*prgH::gfp* reporter fusion were diluted 10 times in distilled water containing either 30 µg/mL propidium iodide (PI) (Invitrogen) or 10 µM Sytox blue (Invitrogen). The mixtures were incubated in 96 well plates for 30 min at 37°C. After incubation, cells were diluted 10 times in filtered PBS and analyzed by flow cytometry using LSR Fortessa (BD Bioscience) operated with the FACS Diva software (BD Bioscience). Data acquisition was performed until 50.000 events corresponding to unstained live cells were recorded using excitation with 561 nm laser and band pass filter 610/20 nm for PI, and excitation with 405 nm laser and band pass filter 450/50 nm for Sytox Blue. The GFP signal was recorded using excitation with 488 nm laser and band pass filter 512/25 nm and 505LP. Data were processed using FlowJo V10 software (FlowJo, LCC). The threshold for determining the proportion of GFP positive cells among unstained cells (PI or Sytox blue negative) was set according to the distribution of fluorescence in the Δ*hilD* population, mainly GFP negative.

### Virulence analysis in mice

Nine- to twelve-week-old specific and opportunistic pathogen free (SOPF) C57BL/6 mice were pretreated with 25 mg of streptomycin by oral gavage 24 h prior to infection with *S.* Typhimurium to allow robust colonization (39). Strains were grown overnight in LB containing the appropriate antibiotics. Prior to infection, the strains were diluted 1:20 in LB without antibiotics and incubated for 4 h at 37°C. Cells were then washed in PBS before oral gavage of the mice. Each 50 µL inoculum contained ca. 5 × 10^7^ CFU. Fecal samples were collected daily, homogenized in 1 mL PBS by bead beating, and bacterial populations were enumerated by selective plating on MacConkey agar containing the appropriate antibiotics. In addition, samples were frozen for determination of the lipocalin 2 concentration. To determine the intensity of the inflammatory in the gut, serial dilutions of fecal samples were analyzed using the Mouse Lipocalin-2/NGAL DuoSet ELISA kit (R&D Systems) according to the manufacturer’s instructions. Bacterial loads in mesenteric lymph nodes were determined by selective plating of the homogenized tissues in PBS containing 0.5% tergitol and 0.5% BSA.

All animal experiments were approved by the legal authorities (Basel-Stadt Kantonales Veterinäramt, licence #30480) and followed the 3R guidelines to reduce animal use and suffering to its minimum.

### Statistical analysis

Statistical analyses were performed using the open-source software R [R Core Team, 2021; (40)], or GraphPad Prism (GraphPad Software, Inc., San Diego, CA, USA). Statistical tests are indicated in figure legends.

## Data Availability

Raw whole genome sequencing reads are available in the NCBI Sequence read archive (SRA) under Bioproject PRJNA1032469.

## SUPPLEMENTAL MATERIAL

Supplemental figures mbio.03105-23-s0001.pdf Figures S1, S2, and S3.

Table S1 mbio.03105-23-s0002.docx All strains and plasmids used in this study.

Table S2 mbio.03105-23-s0003.docx All primers used in this study.

ASM does not own the copyrights to Supplemental Material that may be linked to, or accessed through, an article. The authors have granted ASM a non-exclusive, world-wide license to publish the Supplemental Material files. Please contact the corresponding author directly for reuse.
