# Supplementary material for: Evolutionary trade-off between heat shock resistance, growth at high temperature, and virulence expression in Salmonella Typhimurium
Source: mBio. 2024 Feb 13;15(3):e03105-23. doi: 10.1128/mbio.03105-23 (PMC10936172; doi:10.1128/mbio.03105-23)
Supplement: Supplemental figures — Figures S1, S2, and S3. [file mbio.03105-23-s0001.pdf]

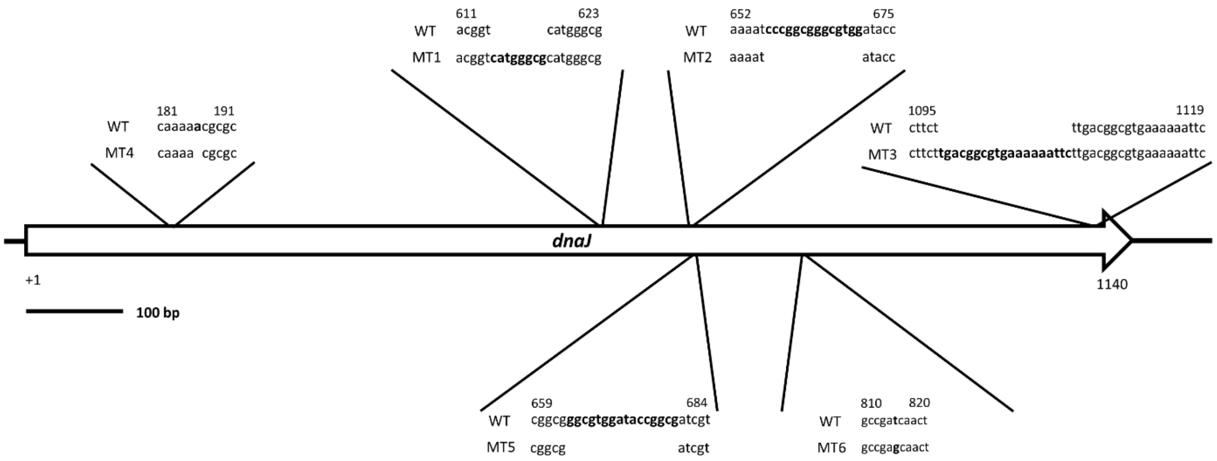

Figure S1: Detailed overview of the mutations observed in the *dnaJ* alleles of the heat resistant mutants (MT1-6) in comparison to the wild type parental (WT) sequence. Numbering starts from the first base of the start codon of the *dnaJ* open reading frame and is indicated for the WT sequence.

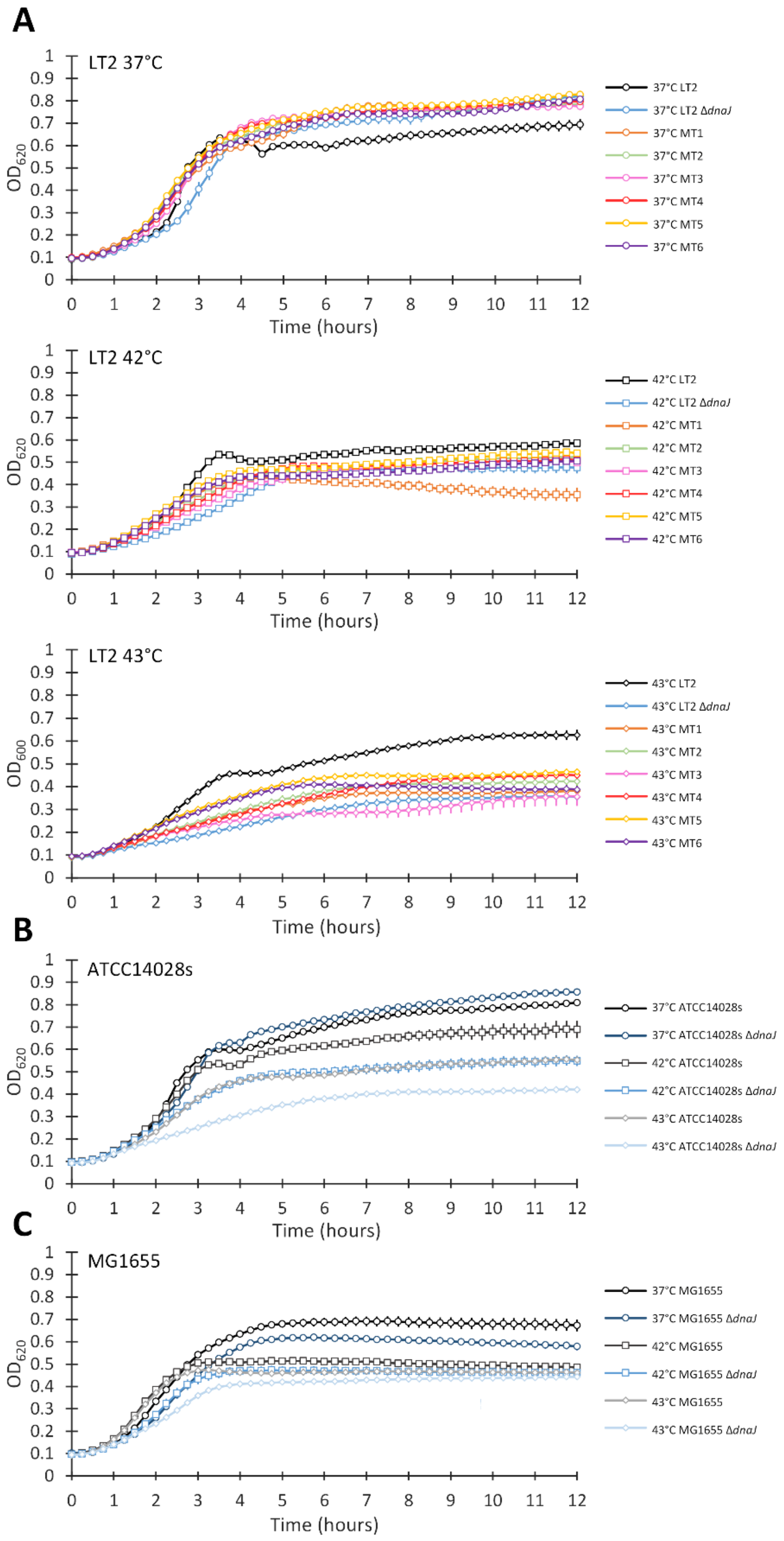
A
LT2 37°C
OD_{620}
Time (hours)
37°C LT2
37°C LT2 Δ*dnaJ*
37°C MT1
37°C MT2
37°C MT3
37°C MT4
37°C MT5
37°C MT6
LT2 42°C
OD_{620}
Time (hours)
42°C LT2
42°C LT2 Δ*dnaJ*
42°C MT1
42°C MT2
42°C MT3
42°C MT4
42°C MT5
42°C MT6
LT2 43°C
OD_{600}
Time (hours)
43°C LT2
43°C LT2 Δ*dnaJ*
43°C MT1
43°C MT2
43°C MT3
43°C MT4
43°C MT5
43°C MT6
B
ATCC14028s
OD_{620}
Time (hours)
37°C ATCC14028s
37°C ATCC14028s Δ*dnaJ*
42°C ATCC14028s
42°C ATCC14028s Δ*dnaJ*
43°C ATCC14028s
43°C ATCC14028s Δ*dnaJ*
C
MG1655
OD_{620}
Time (hours)
37°C MG1655
37°C MG1655 Δ*dnaJ*
42°C MG1655
42°C MG1655 Δ*dnaJ*
43°C MG1655
43°C MG1655 Δ*dnaJ*

Figure S2: (A) Optical densities (measured at 620 nm) of *S.* Typhimurium LT2*,* its Δ*dnaJ* derivative, and the heat-selected mutants (MT1-6) grown for 12 hours in TSB microtiter plates at 37°C (top), 42°C (middle) and 43°C (bottom). (B) Optical densities (620 nm) of *S.* Typhimurium ATCC14028s and its Δ*dnaJ* derivative grown for 12 hours in TSB microtiter plates at 37°C, 42°C and 43°C. (C) Optical densities (620 nm) of *E. coli* MG1655 Δ*lacY* and its Δ*dnaJ* derivative grown for 12 h in TSB microtiter plates at 37°C, 42°C and 43°C. For all panels, error bars indicate the standard error over 6 separately grown cultures.

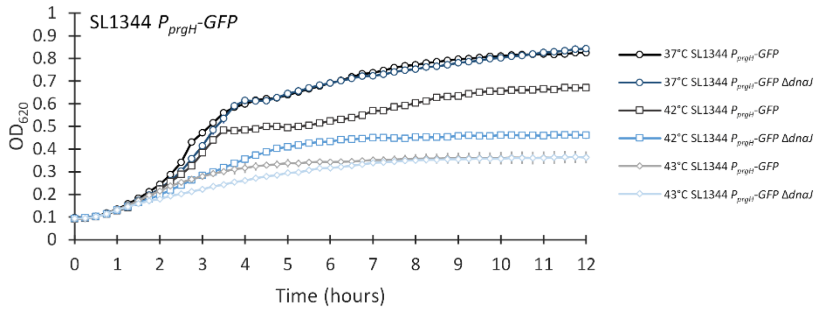

Figure S3: Optical densities (620 nm) of *S.* Typhimurium SL1344 P_{prgH}-*GFP* wild type and its Δ*dnaJ* derivative grown for 12 h in TSB microtiter plates at 37°C, 42°C and 43°C. Error bars indicate the standard error over 6 separately grown cultures.
