# Supplementary material for: Evolutionary trade-off between heat shock resistance, growth at high temperature, and virulence expression in Salmonella Typhimurium
Source: mBio. 2024 Feb 13;15(3):e03105-23. doi: 10.1128/mbio.03105-23 (PMC10936172; doi:10.1128/mbio.03105-23)
Supplement: Table S1 — All strains and plasmids used in this study. [file mbio.03105-23-s0002.docx]

Supplementary Table 1. Overview of strains and plasmids used in this study.

| **Strain** | **Description** | **Source or reference** |
| --- | --- | --- |
| *Salmonella enterica* Typhimurium | | |
| LT2 | *S.* Typhimurium LT2 wild type | Spanish Type Culture Collection |
| MT1 - MT6 | Heat resistant *S.* Typhimurium strains obtained from evolution lineages 1, 2, 3, 4, 5 and 6, respectively. | This study |
| LT2 Δ*dnaJ* | *S.* Typhimurium LT2 in which the *dnaJ* open reading frame is replaced with an *frt*-flanked *nptII* cassette | This study |
| ATCC14028s | *S.* Typhimurium ATCC14028s wild type | American Type Culture Collection |
| ATCC14028s Δ*dnaJ* | *S.* Typhimurium ATCC14028s in which the *dnaJ* open reading frame is replaced with an *frt*-flanked *nptII* cassette | This study |
| SL1344 *P_prgH_-GFP* | *S.* Typhimurium SL1344 carrying a transcriptional fusion of *GFP* to the *prgH* promoter | [41] |
| SL1344 *P_prgH_-GFP* Δ*dnaJ* | *S.* Typhimurium SL1344 *P_prgH_-GFP* in which the *dnaJ* open reading frame is replaced with an *frt*-flanked *nptII* cassette | This study |
| SL1344 *P_prgH_-GFP* Δ*hilD* | *S.* Typhimurium SL1344 *P_prgH_-GFP* with an in-frame deletion of *hilD* | [41] |
| SL1344 *P_prgH_-GFP* Δ*hilE* | *S.* Typhimurium SL1344 *P_prgH_-GFP* with an in-frame deletion of *hilE* | [41] |
| SL1344 *P_prgH_-GFP* Δ*hilD*  Δ*dnaJ* | *S.* Typhimurium SL1344 *P_prgH_-GFP* Δ*hilD* in which the *dnaJ* open reading frame is replaced with an *frt*-flanked *nptII* cassette | This study |
| SL1344 *P_prgH_-GFP* Δ*hilE*  Δ*dnaJ* | *S.* Typhimurium SL1344 *P_prgH_-GFP* Δ*hilE* in which the *dnaJ* open reading frame is replaced with an *frt*-flanked *nptII* cassette | This study |
| *Escherichia coli* | | |
| MG1655 Δ*lacY* | *E. coli* MG1655 with an in-frame deletion of *lacY* | [19] |
| MG1655 Δ*lacY* Δ*dnaJ* | *E. coli* MG1655 with in-frame deletions of *lacY* and *dnaJ* | This study |
| **Plasmid** | **Description** | **Source or reference** |
| pKD46 | Encodes lambda red genes under control of an arabinose-inducible promoter | [36] |
| pCP20 | Encodes Flp for recombining *frt* sites (to excise sequences in between) | [37] |
|  |  |  |

References:

41. Sturm A, Heinemann M, Arnoldini M, Benecke A, Ackermann M, Benz M, Dormann J, Hardt W-D. 2011. The cost of virulence: retarded growth of *Salmonella* Typhimurium cells expressing type iii secretion system 1. PLoS Pathog 7:e1002143. <https://doi.org/10.1371/journal.ppat.1002143>
