# Supplementary material for: Evolutionary trade-off between heat shock resistance, growth at high temperature, and virulence expression in Salmonella Typhimurium
Source: mBio. 2024 Feb 13;15(3):e03105-23. doi: 10.1128/mbio.03105-23 (PMC10936172; doi:10.1128/mbio.03105-23)
Supplement: Table S2 — All primers used in this study. [file mbio.03105-23-s0003.docx]

Supplementary Table 2. Overview of primers used in this study. When relevant, primer attachment sites are indicated in bold.

| **Name** | **Sequence** | **Description** |
| --- | --- | --- |
| P1 | GGTTTCCTCTCCGCCCGTGTATGCATGTTAAGGGCAGATAAAAAGAGATGATTCCGGGGATCCGTCGACC | Amplification of the *frt-nptII-frt* cassette from pKD13 for deleting the *dnaJ* gene in LT2 |
| P2 | GGGCTGAAGAAAAATACAACGGGAAAAGATTAGCGAGTCAAATCGTCAAATGTAGGCTGGAGCTGCTTCG | Amplification of the *frt-nptII-frt* cassette from pKD13 for deleting the *dnaJ* gene in LT2 |
| P3 | GATAAAGCCGCTATCGAAGC | Control and sequencing of *dnaJ* deletion in LT2 |
| P4 | TAATGTCATCACATCACGTT | Control and sequencing of *dnaJ* deletion in LT2 |
| P5 | GGTTTCCTCTCCGCCCGTGTATGCATGTTAAGGGCAGATAAAAAGAGATG**GTGTAGGCTGGAGCTGCTT** | Amplification of the *frt-nptII-frt* cassette from pKD13 for deleting the *dnaJ* gene in ATCC14028s |
| P6 | GGGCTGAAGAAAAATACAACGGGAAAAGATTAGCGAGTCAAATCGTCAAA**GATCCGTCGACCTGCAGTTC** | Amplification of the *frt-nptII-frt* cassette from pKD13 for deleting the *dnaJ* gene in ATCC14028s |
| P7 | TGCAAACAACGCGAAAGATGA | Control and sequencing of *dnaJ* deletion in ATCC14028s |
| P8 | GAATAAGCGTATACAAAACTGCTGATTAAC | Control and sequencing of *dnaJ* deletion in ATCC14028s |
| P9 | GAATTTCCTCTCCGCCCGTGCATTCATCTAGGGGCAATTTAAAAAAGATG**GTGTAGGCTGGAGCTGCTT** | Amplification of the *frt-nptII-frt* cassette from pKD13 for deleting the *dnaJ* gene in MG1655 |
| P10 | GCCTGCCCACGGGCAGGCTTTTGGGGAGGTTAGCGGGTCAGGTCGTCAAA**GATCCGTCGACCTGCAGTTC** | Amplification of the *frt-nptII-frt* cassette from pKD13 for deleting the *dnaJ* gene in MG1655 |
| P11 | CTGCCGGTGCTGATG | Control and sequencing of *dnaJ* deletion in MG1655 |
| P12 | GGCGTAATACCACAACCC | Control and sequencing of *dnaJ* deletion in MG1655 |
| P13 | GGCAGATAAAAAGAGATGGCGAAAAGAGATTACTACGAGATGTGTGTAGGCTGGAGCTGCTTC | Amplification of the *frt-nptII-frt* cassette from pKD13 for deleting the *dnaJ* gene in SL1344 |
| P14 | TACAACGGGAAAAGATTAGCGAGTCAAATCGTCAAAGAATCATCATATGAATATCCTCCTTAG | Amplification of the *frt-nptII-frt* cassette from pKD13 for deleting the *dnaJ* gene in SL1344 |
| P15 | ATCGCTCAGCAGCAACATG | Control and sequencing of *dnaJ* deletion in SL1344 |
| P16 | TATACAAAACTGCTGATTAACATC | Control and sequencing of *dnaJ* deletion in SL1344 |
|  |  |  |
